# Safety and Reactogenicity of Canarypox ALVAC-HIV (vCP1521) and HIV-1 gp120 AIDSVAX B/E Vaccination in an Efficacy Trial in Thailand

**DOI:** 10.1371/journal.pone.0027837

**Published:** 2011-12-21

**Authors:** Punnee Pitisuttithum, Supachai Rerks-Ngarm, Valai Bussaratid, Jittima Dhitavat, Wirach Maekanantawat, Swangjai Pungpak, Pravan Suntharasamai, Sirivan Vanijanonta, Sorachai Nitayapan, Jaranit Kaewkungwal, Michael Benenson, Patricia Morgan, Robert J. O'Connell, Jeffrey Berenberg, Sanjay Gurunathan, Donald P. Francis, Robert Paris, Joseph Chiu, Donald Stablein, Nelson L. Michael, Jean-Louis Excler, Merlin L. Robb, Jerome H. Kim

**Affiliations:** 1 Faculty of Tropical Medicine, Mahidol University, Bangkok, Thailand; 2 Department of Disease Control, Ministry of Public Health, Bangkok, Thailand; 3 Armed Forces Research Institute of Medical Sciences, Bangkok, Thailand; 4 U.S. Military HIV Research Program, Walter Reed Army Institute of Research, Rockville, Maryland, United States of America; 5 Department of Medicine, Tripler Army Medical Center, Honolulu, Hawaii, United States of America; 6 Sanofi Pasteur, Swiftwater, Pennsylvania, United States of America; 7 Global Solutions for Infectious Diseases, South San Francisco, California, United States of America; 8 Walter Reed Army Medical Center, Washington, D. C., United States of America; 9 Division of AIDS, National Institute of Allergy and Infectious Diseases, National Institutes of Health, Bethesda, Maryland, United States of America; 10 EMMES Corporation, Rockville, Maryland, United States of America; University of Sao Paulo, Brazil

## Abstract

**Background:**

A prime-boost vaccination regimen with ALVAC-HIV (vCP1521) administered intramuscularly at 0, 4, 12, and 24 weeks and gp120 AIDSVAX B/E at 12 and 24 weeks demonstrated modest efficacy of 31.2% for prevention of HIV acquisition in HIV-uninfected adults participating in a community-based efficacy trial in Thailand.

**Methodology/Principal Findings:**

Reactogenicity was recorded for 3 days following vaccination. Adverse events were monitored every 6 months for 3.5 years, during which pregnancy outcomes were recorded. Of the 16,402 volunteers, 69% of the participants reported an adverse event any time after the first dose. Only 32.9% experienced an AE within 30 days following any vaccination. Overall adverse event rates and attribution of relatedness did not differ between groups. The frequency of serious adverse events was similar in vaccine (14.3%) and placebo (14.9%) recipients (p = 0.33). None of the 160 deaths (85 in vaccine and 75 in placebo recipients, p = 0.43) was assessed as related to vaccine. The most common cause of death was trauma or traffic accident. Approximately 30% of female participants reported a pregnancy during the study. Abnormal pregnancy outcomes were experienced in 17.1% of vaccine and 14.6% (p = 0.13) of placebo recipients. When the conception occurred within 3 months (estimated) of a vaccination, the majority of these abnormal outcomes were spontaneous or elective abortions among 22.2% and 15.3% of vaccine and placebo pregnant recipients, respectively (p = 0.08). Local reactions occurred in 88.0% of vaccine and 61.0% of placebo recipients (p<0.001) and were more frequent after ALVAC-HIV than AIDSVAX B/E vaccination. Systemic reactions were more frequent in vaccine than placebo recipients (77.2% vs. 59.8%, p<0.001). Local and systemic reactions were mostly mild to moderate, resolving within 3 days.

**Conclusions/Significance:**

The ALVAC-HIV and AIDSVAX B/E vaccine regimen was found to be safe, well tolerated and suitable for potential large-scale use in Thailand.

**Trial Registration:**

ClinicalTrials.gov NCT00223080

## Introduction

HIV/AIDS has emerged as a worldwide public health threat and is associated with high morbidity and mortality. Worldwide, the total number of people living with HIV in 2009 was estimated to be 33.3 million with 2.6 million being newly infected [1]. In Thailand, 14,000 new HIV infections occur each year despite the considerable efforts and success in controlling the HIV epidemic [2], [3]. The circulating recombinant form CRF01_AE and subtype B dominate the HIV epidemic in Thailand [4], [5]. The development of a safe, effective, easily administered and inexpensive AIDS vaccine is desperately needed worldwide, and the Thai Ministry of Public Health has long recognized that need and has strongly supported HIV vaccine research in Thailand [6], [7]. An AIDS vaccine as part of a comprehensive prevention package is considered the best long-term solution in controlling the HIV/AIDS pandemic [8], [9]. Safety is a paramount consideration for all preventive vaccines. Monitoring and assessing vaccine safety is a priority for public health. It is generally thought that such interventions must have modest rates of reactions and only rare severe or serious events associated with their use [10], [11].

This prime-boost concept applied to AIDS vaccines employs viral vector prime together with a soluble envelope subunit boost. The concept is aimed specifically at inducing both CD4+ and CD8+ T-cell as well as binding and neutralizing antibody immune responses [12]–[15]. An effective immune response will likely comprise a combination of antibodies and CD4+ and CD8+ T cells that recognize, neutralize and/or destroy diverse strains of HIV before an infection becomes irreversibly established [16]. Given the hurdles of eliciting broadly neutralizing antibodies, the focus of HIV vaccine development in recent years turned to evaluating vaccines capable of reducing viral replication after infection (“T-cell vaccines”) [17], [18]. Although contradicted by some studies [19], control of viral replication could conceivably slow the rate of disease progression as suggested by non-human primate (NHP) challenge studies [20]–[23] and/or reduce transmission of HIV from infected vaccine recipient to partner [24].

Earlier HIV vaccine trials from 1994–2000 tested recombinant protein candidate vaccines that were capable of inducing antibody responses [25]. One of these, a bivalent recombinant gp120 (AIDSVAX B/E) derived from HIV-1 CRF01_AE and B subtypes was tested in Phase I/II trials and was shown to be safe and immunogenic [26], [27]. A Phase III trial using AIDSVAX B/E in Thai injecting drug users, while confirming safety, did not provide evidence of protection against HIV acquisition. In addition, a concurrent Phase III trial using a bivalent gp120 subtype B vaccine (AIDSVAX B/B′), among North American and European men who have sex with men and women at high risk for heterosexual transmission of HIV, did not protect against HIV infection [28], [29].

Attenuated non-replicating poxvirus vectors, in particular canarypox (ALVAC) vectors, have been extensively studied and appeared to be safe in phase I and II clinical studies [30]. ALVAC prime and recombinant gp160 or gp120 boosts induced cell-mediated immune responses together with significantly enhanced antibody responses in HIV-uninfected volunteers. These initial clinical studies conducted on a limited number of healthy subjects in various parts of the world have documented the safety profile of these two vaccines alone or combined in prime-boost regimen [31]–[42]. In Thailand, phase I/II trials of ALVAC-HIV (vCP1521) and several recombinant gp120 or gp160 boosts showed that the vaccines were well tolerated and immunogenic [27], [43], [44]. These preliminary studies led to efficacy testing of this prime-boost regimen in a large (>16,000 persons) trial initiated in 2003 in Rayong and Chon Buri provinces of Thailand. The trial demonstrated a modest efficacy of 31.2% for prevention of HIV acquisition compared to placebo in a modified intention-to-treat analysis [45]. This prime-boost regimen had demonstrated an adequate safety profile in previous human studies that allowed advancement to full-scale efficacy testing. These large-scale, randomized, controlled efficacy studies provide the most valid, time-tested approach for evaluation of adverse events that may be related to vaccination in the targeted population. This paper presents the safety and tolerability profile of ALVAC-HIV and AIDSVAX B/E in 16,402 volunteers participating in the RV144 Thai Phase III HIV vaccine study.

## Methods

### Study Setting

The study was conducted through facilities of the Thai Ministry of Public Health in Rayong and Chon Buri provinces. The protocol for this trial and supporting CONSORT checklist are available as supporting information; see Checklist S1 and Protocol S1. This trial is registered at clinicaltrials.gov, registry number NCT00223080.

### Participants

Eligible HIV-uninfected male and female adults aged 18–30 years from the general population in Rayong and Chon Buri provinces of Thailand were enrolled and randomly assigned to vaccine or placebo. Pregnant and breastfeeding women were excluded from trial participation. Female participants were advised to practice effective birth control and avoid pregnancy until 3 months after the last vaccination [45].

### Ethical Compliance

The protocol was reviewed by the ethics committees of the Ministry of Public Health, the Royal Thai Army, Mahidol University, and the Human Subjects Research Review Board of the U.S. Army Medical Research and Materiel Command. It was also independently reviewed and endorsed by the World Health Organization and the Joint United Nations Program on HIV/AIDS and by the AIDS Vaccine Research Working Group of the National Institute of Allergy and Infectious Diseases at the National Institutes of Health [45]. All participants provided their written informed consent. An independent Data and Safety Monitoring Board conducted periodic reviews for safety, futility, and efficacy.

### Interventions

RV144 was a community-based, multicenter, randomized, double blind, placebo-controlled efficacy trial of the recombinant canarypox vector vaccine ALVAC-HIV (vCP1521) and recombinant gp120 AIDSVAX B/E administered in a prime-boost vaccination regimen [45]. Eligible participants received ALVAC-HIV (vCP1521) (Sanofi Pasteur, Swiftwater, PA, USA) or placebo at weeks 0, 4, 12 and 24 and AIDSVAX B/E (Global Solutions for Infectious Diseases, South San Francisco, CA, USA) or placebo at weeks 12 and 24. ALVAC-HIV is a live recombinant canarypox vector vaccine that has been genetically engineered to express subtype E HIV-1 gp120 (strain 92TH023) linked to the transmembrane anchoring portion of gp41 (strain LAI), and HIV-1 gag and protease (LAI strain). ALVAC-HIV is grown in chicken embryo fibroblasts and formulated at a dose of 10^6^ CCID50 with 10 mM Tris HCl, pH 9 and lactoglutamate. ALVAC-HIV is formulated as a lyophilized vaccine for injection and is reconstituted with 1.0 mL of sterile sodium chloride (0.4% NaCl) for a single dose. AIDSVAX B/E vaccine is a highly purified mixture of gp120 proteins produced by recombinant DNA procedures using Chinese hamster ovary (CHO) cell expression. The sequences of MN gp120/HIV-1 and A244 gp120/HIV-1 are expressed as fusion proteins where a 27 amino acid sequence of the herpes simplex virus type 1 gD protein is fused to the amino terminus of each protein. MN and A244 rgp120/HIV-1 are combined to produce the bivalent AIDSVAX B/E vaccine. AIDSVAX B/E is supplied as a sterile suspension in single-use glass vials. Each vial has a nominal content of 1 mL (300 µg/mL) of each rgp120/HIV-1 protein adsorbed onto a total of 600 µg aluminum hydroxide gel adjuvant. ALVAC placebo was a sterile, lyophilized preparation consisting of a virus stabilizer in 1 mL of 0.4% sodium chloride while AIDSVAX placebo consisted of 600 µg aluminum hydroxide gel adjuvant. ALVAC-HIV or placebo was administered in the deltoid muscle of the left arm and AIDSVAX B/E or placebo in the deltoid muscle of the right arm. Female volunteers were vaccinated only if a urine pregnancy test was negative the day of the vaccination visit.

### Objectives

The primary objective of this analysis was to evaluate the safety and reactogenicity of ALVAC-HIV and recombinant gp120 AIDSVAX B/E administered in a prime-boost vaccination regimen. The results of vaccine efficacy and immunogenicity have been published elsewhere [45].

### Outcomes

Local reactions were separately recorded for each of the ALVAC-HIV or placebo and AIDSVAX B/E or placebo injections since they were administered in separate arms. Selected adverse events monitored included local reactions at the injection site - namely erythema, induration (mild: 1–9 mm; moderate: 10–19 mm; severe: >20 mm), pain and tenderness, swelling and limitation of arm movement and the systemic reactions of fever (oral temperature ≥37.8°C), tiredness, myalgia, arthralgia, headache, rash and nausea, vomiting and are hereafter termed “*post vaccination reactions*”. Systemic reactions following the third and fourth vaccinations could not be attributed to the ALVAC-HIV or AIDSVAX B/E separately as both were administered at the same time. Systemic events that had a clearly recognized cause not related to the vaccination (for example dengue fever) were not reported as “*post-vaccination reactions*”.

Reactogenicity was self-reported for 3 days on diary cards, which were reviewed by the nurse and the volunteer at the next visit. If the nurse observed inconsistencies, the volunteer would correct the card and the corrected information then recorded into the case report form. If there were blanks on the card or the volunteer could not remember, the volunteer was not allowed to fill in the card from memory and was instructed to put a dash for the value.

If unusual or severe signs or symptoms occurred after vaccination, subjects were instructed by study personnel to seek medical attention within the district where they were vaccinated or at another Ministry of Public Health facility. Staff at health centers referred volunteers to the district hospital for further evaluation and treatment as appropriate. These subjects, if possible, were followed up clinically until resolution of symptoms.

An adverse event (AE) was defined as any undesired, noxious or pathological change in participants as indicated by physical signs, symptoms, and/or laboratory changes that occurred following administration of one of the vaccines, whether or not considered vaccine-related. This definition included intercurrent illnesses or injuries, and unexpected exacerbations of pre-existing conditions. Anticipated day-to-day fluctuations of pre-existing conditions that did not represent a clinically significant exacerbation were not considered adverse events. Discrete episodes of chronic conditions occurring during the study period were reported as adverse events in order to assess changes in frequency or severity.

All adverse events occurring up to week 54 (20 weeks post last vaccination) that resulted in an encounter with a health care provider (physician, nurse, etc) were elicited, recorded on source documents and transcribed onto case report forms (CRF). After week 54, and up to week 184, only AE's that were “medically significant”, defined as requiring multiple visits (two or more) to a physician for the same condition, or that resulted in hospitalization or an emergency room visit, were captured on source documents and CRF. Medications were reported in association with all AE. Data on serious adverse events (SAE as per definition of the U.S. Food and Drug Administration) occurring through the whole period of the study were collected and recorded on CRF, as well as reported separately on SAE report forms. A subject with an SAE was followed carefully until the condition resolved or stabilized and/or chronicity was established. Any medication or other therapeutic measure taken to relieve symptoms of the medical problem was recorded on the CRF with the report of the outcome on the SAE forms. Deaths were recorded and their causes determined to the extent possible.

All AEs, including reactogenicity events, were graded as mild, moderate and severe as recommended by the Division of Acquired Immunodeficiency Syndrome of the National Institutes of Allergy and Infectious Diseases, and categorized according to the Medical Dictionary for Regulatory Activities (MedDRA) organ class system. Relationship to vaccination was established by a study physician at the time of reporting without knowledge of treatment assignment

Pregnancy outcomes were recorded, including spontaneous and induced abortions. Pregnancy outcome information was obtained from hospital records when available or from the volunteer. The time from estimated conception to the last vaccination prior to pregnancy diagnosis was calculated.

### Sample Size

Sample size for this efficacy trial was designed to detect a vaccine-associated 25% decrease in the hazard rate during the vaccination period and 50% in the subsequent 3 years. A placebo arm infection rate of 0.34%/year was assumed using the lower bound of the 95% confidence interval from a field study in Chon Buri of 20–30 year olds. With up to 5% losses to follow-up per 6 month period, a total sample of 16,000 subjects provided 90% power using a two-sided 5% Type 1 error rate, to detect vaccine efficacy greater than zero. Event rate differences greater than 3% can be detected with >90% power when the true rates are near 50%.

### Randomization and Blinding

Randomization used centrally (EMMES Corporation) generated permuted blocks of random sizes for a set of coded treatment labels that coincided with coded treatment stocks. Study pharmacists, independent of other site staff and blinded to the contents of the coded treatment stocks, maintained the randomization lists and prepared opaque syringes for clinic staff. Study site staff, volunteers, and laboratories remained blinded with respect to the allocation of placebo or vaccine.

### Statistical Methods

Demographic and safety comparisons included all volunteers in an intention-to-treat analysis. Reactogenicity, adverse events and serious adverse events were tabulated both overall and by study arm. Proportions experiencing reactions overall and after individual vaccinations along with product-limit estimates of time to adverse and serious adverse events were computed. Frequencies of specific safety events and pregnancy outcomes were compared across study arms using a chi-square test to evaluate the null hypothesis that safety event rates are the same in both study arms and the Wilcoxon test was used to compare severity grades. Odds ratios were estimated with logistic regression and p values <0.05 were considered significant.

## Results

### Participant flow

The CONSORT flow diagram of the trial has been published elsewhere [45]. A total of 16,402 study volunteers were randomized (8,202 and 8,200 vaccine and placebo recipients, respectively), including 10,068 (61.4%) male participants. Of the total, 14,802 completed the study. Seven volunteers (5 vaccine and 2 placebo recipients) were HIV-infected at baseline. Six randomized cases did not receive the initial vaccination and 13,973 (85.2%) received all four doses of vaccine or placebo. A total of 1,593 (9.7%) volunteers discontinued visits from the study: 796 (9.7%) from vaccine and 797 (9.7%) in placebo group. The reasons included: loss to follow-up, refusal of further participation, geographic relocation, death, and unknown reasons.

### Recruitment

The study lasted from October 2003 to June 2009. The demographic characteristics of the vaccine and placebo groups are shown in Table 1.

**Table 1:** Demographic characteristics of RV144 participants in vaccine and placebo groups.

		Vaccine (n = 8,202)	Placebo (n = 8,200)	Total (n = 16,402)
		n (%)	n (%)	n (%)
Sex	Male	5,037 (61.4)	5,031 (61.4)	10,068 (61.4)
	Female	3,165 (38.6)	3,169 (38.6)	6,334 (38.6)
Age Years	≤20	2,300 (28)	2,246 (27.4)	4,546 (27.7)
	21–25	3,635 (44.3)	3,709 (45.2)	7,344 (44.8)
	≥26	2,267 (27.7)	2,245 (27.4)	4,512 (27.5)

### Reactogenicity

Reaction reporting was typically complete and bounded by no more than 5% loss for each time point requested. For example, after the first vaccination, 4.2% of the volunteers were missing the 6-hour local reaction assessments, which decreased to 1.7% at the 72-hour assessment. A majority of participants experienced local and systemic reactions, but more occurred in vaccine recipients (7,442; 91.9%) as compared to placebo (6,141; 75.7%) recipients (p<0.001). We examined reaction rates after the first dose in participants who returned to receive subsequent doses comparatively to those who discontinued vaccinations. Overall reactogenicity rates were higher in participants who discontinued comparatively to those who returned (90.5% vs. 86.9% in vaccine recipients, respectively, p = 0.021; 64.5% vs. 56.0% in placebo recipients, respectively, p<0.001).

#### Local reactogenicity

Considering all doses administered, local reactions were more frequently observed in vaccine (ALVAC-HIV and/or AIDSVAX B/E) (7,125; 88.0%) than in placebo recipients (4,942; 61.0%) (p<0.001). Local reactions were more common after the first dose (81.3% for vaccine recipients vs. 32.5% for placebo recipients, p<0.001), and reaction rates were lower with subsequent doses (60.8% vs. 24% after second dose, p<0.001).

ALVAC-HIV induced a higher frequency of reactions (87.8%) than AIDSVAX B/E (54.6%) (p<0.001) (Table 2). For both products, pain and/or tenderness were the most frequent local reactions observed, followed by arm movement limitation. All reactions were generally mild and transient, resolving within 3 days. The proportion of participants with individual local reaction types, with the exception of induration and erythema (<4% for ALVAC-HIV and <1% for AIDSVAX B/E), was significantly higher in the vaccine group than in the placebo group (p<0.001) for all types of reactions (Table 2).

**Table 2:** Characteristics and overall frequency of local reactions in vaccine and placebo groups.

	ALVAC – HIV				AIDSVAX® B/E			
	Vaccine		Placebo		Vaccine		Placebo	
	n = 8,096		n = 8,107		n = 7,159		n = 7,262	
	n	%	n	%	n	%	n	%
Pain/Tenderness	6,852	84.6	3,727	46.0	3,543	49.5	2,991	41.2
Arm movement limitation	5,647	69.8	2,129	26.3	2,627	36.7	1,943	26.8
Swelling	2,325	28.7	432	5.3	643	9.0	379	5.2
Erythema >0 mm	283	3.5	177	2.2	70	1.0	72	1.0
Induration >0 mm	316	3.9	108	1.3	60	0.8	53	0.7
Any local reaction	7,107	87.8*	4,210	51.9	3,908	54.6*	3,364	46.3

*p<0.001.

The proportion of participants with individual local reaction types, with the exception of induration and erythema, was significantly higher in the vaccine group than in the placebo group (p<0.001) for all types of reactions.

#### Systemic reactogenicity

Participants reported systemic reactions more frequently in the combined vaccine group (6,252; 77.2%) than in the placebo group (4,850; 59.8%) (p<0.001). The characteristics and frequency of systemic reactions reported are shown in Table 3. Fatigue, myalgia, headache, and arthralgia were the most common reactions reported in both study arms and were significantly higher in vaccine than in placebo recipients (p<0.001). Rash was reported infrequently (<5%) and at similar rates by both vaccine and placebo recipients. Symptoms typically resolved within 3 days.

**Table 3:** Characteristics and overall frequency of systemic reactions in vaccine or placebo groups.

	Vaccine (n = 8,096)		Placebo (n = 8,107)	
	N	%	n	%
Headache	3,677	45.4	2,522	31.1
Fatigue	5,182	64.0	3,410	42.1
Myalgia	4,237	52.3	2,494	30.8
Arthralgia	2,177	26.9	1,246	15.4
Oral temperature ≥37.8°C	1,564	19.3	1,009	12.4
Nausea/Vomiting	1,080	13.3	802	9.9
Any reaction	6,251	77.2%*	4,850	59.8%

*p<0.001.

Systemic reactions following the third and fourth vaccinations could not be attributed to either the ALVAC-HIV or AIDSVAX B/E separately as both were administered simultaneously although in two different arms.

#### Severity of reactions

**Figure 1:**
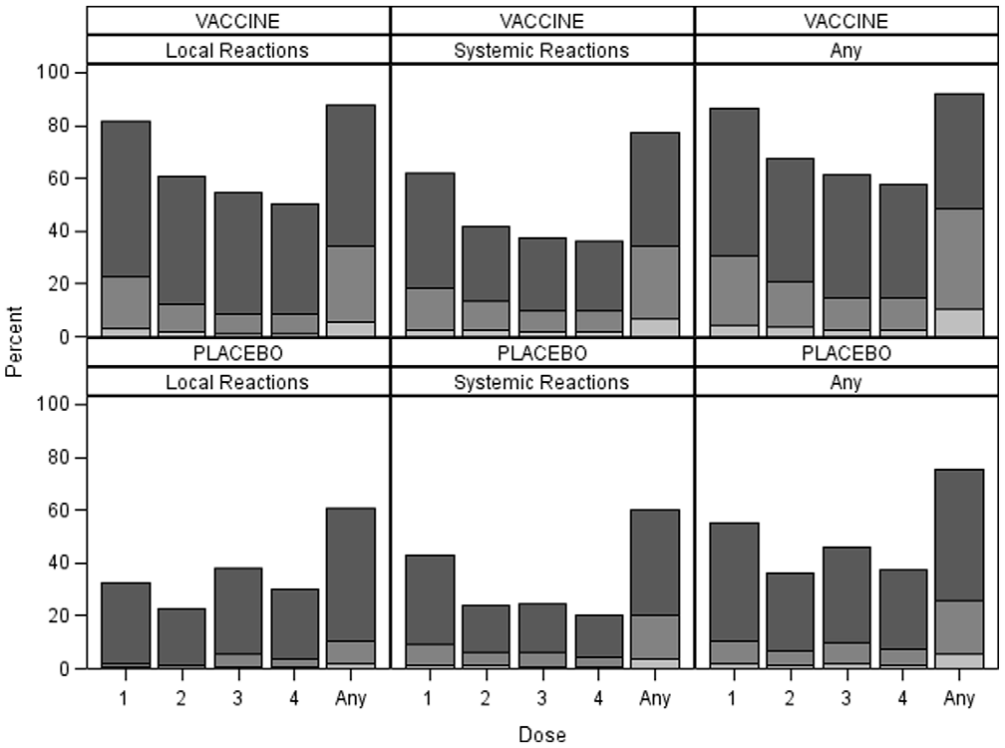
Percentage of individuals with local and systemic reactions by treatment and dose administered. Local reactions were separately recorded for each of the ALVAC-HIV or placebo and AIDSVAX B/E or placebo injections since they were administered in separate arms. Systemic reactions following the third and fourth vaccinations could not be attributed to the ALVAC-HIV or AIDSVAX B/E separately as both were administered simultaneously, although in two different arms. Severe (dark grey), moderate (mild grey) and mild (light grey).

As shown in Figure 1, local and systemic reactions were mostly mild to moderate in severity. However, moderate to severe reactions were significantly more common (3,904; 48.2%) in vaccine than in placebo recipients (2,101; 25.9%) (p<0.001) and in ALVAC-HIV (2,706; 33.4%) than AIDSVAX B/E (634; 8.8%) recipients (p<0.001)(not shown).

### Adverse Events

Overall, 69% (11,310) of the participants reported an AE any time on study after the first dose administration, while only 32.9% (5,394) experienced an AE within 30 days after any vaccination. A total of 27,657 episodes of AEs was reported: 13,692 in vaccine and 13,965 in placebo groups. The AE rate in the month post vaccination declined from 15.9% after the first administration to 11.0% after the second and 8.6% after the last two vaccinations. The proportion of subjects experiencing an AE after any vaccination was not significantly different in vaccine and placebo recipients (p = 0.197) as shown in Table 4. Overall, the odds ratio for an increased AE rate with vaccination was 0.97 (95% CI 0.90–1.03). At 6, 12 and 42 months the estimated AE rates for the combined treatment arms were 47.7%, 57% and 71.5%. The AE rates were significantly different (p<0.001) for both age and sex in both vaccine and placebo groups (Table 5).

**Table 4:** Frequency and rates of participants with Adverse Events and Serious Adverse Events (as per definition of the U.S. Food and Drug Administration) occurring within 30 days post each vaccination, during any 30-day post vaccination interval and any time post first vaccination in vaccine and placebo groups.

	Adverse Events						Serious Adverse Events					
	Vaccine		Placebo		Total		Vaccine		Placebo		Total	
	(n = 8,202)		(n = 8,200)		(n = 16,402)		(n = 8,202)		(n = 8,200)		(n = 16,402)	
Dose	n	%	n	%	n	%	n	%	n	%	n	%
**1**	1,277	15.6	1,336	16.3	2,613	15.9	44	0.5	46	0.6	90	0.5
**2**	816	10.7	860	11.2	1,676	11.0	27	0.4	33	0.4	60	0.4
**3**	614	8.6	597	8.2	1,211	8.4	37	0.5	39	0.5	76	0.5
**4**	599	8.6	630	8.9	1,229	8.8	21	0.3	35	0.5	56	0.4
**Any 30- day post vaccination interval**	2,658	32.4	2,736	33.4	5,394	32.9	126	1.5	150	1.8	276	1.7
**Any time post 1**	5,625	68.6	5,685	69.4	11,310	69.0	1,175	14.3	1,219	14.9	2,394	14.6

Row percentages are based on the number of individuals receiving the specified dose.

**Table 5:** Frequency of adverse (AE) and serious adverse events (SAE) by sex and age range in vaccine and placebo groups.

		Vaccine			Placebo		
		n	AE (%)	SAE (%)	n	AE (%)	SAE (%)
**Sex**	**Male**	5,037	65.9	13.6	5,031	67.7	13.6
	**Female**	3,165	72.9	15.5	3,169	72.0	16.9
**Age**	**≤ 20 years**	2,300	64.4	14.3	2,246	66.0	14.5
	**21–25 years**	3,635	69.1	14.6	3,709	70.3	15.4
	**≥26 years**	2,267	72.0	13.9	2,245	71.1	14.4

AE rates differ by age and sex, SAE rates by sex, p<0.001.

The most common AE categories were ‘infection and infestation’ in 38.8%, followed by ‘injury, poisoning and procedural related complications’ in 31.0% of the participants; results were comparable in both vaccine and placebo groups.

Although many of the events (49%) in each group were mild in severity, the combination of moderate (35%), severe (15%) and potentially life-threatening (<1%) events accounted for nearly 50% of AEs in each group. A limited and similar number of deaths (<1%) were reported in both groups during the study. The majority of all events (95%) in both groups were assessed as not related to vaccination and 97% required no change in the vaccine schedule and resolved. Further characteristics of the events are described in Table 6.

**Table 6:** Characteristics and frequency of adverse events and serious adverse events occurring any time after the first dose in vaccine and placebo groups.

		Adverse Events				Serious Adverse Events			
		Vaccine		Placebo		Vaccine		Placebo	
		(n = 13,692)		(n = 13,965)		(n = 1,428)		(n = 1,484)	
		n	%	n	%	n	%	n	%
**Intensity**	Mild	6,674	48.7	6,835	48.9	3	0.2	5	0.3
	Moderate	4,769	34.8	4,868	34.9	59	4.1	74	5.0
	Severe/Life-threatening	2,164	15.8	2,187	15.7	1,281	89.7	1,330	89.6
	Death	85	0.6	75	0.5	85	6.0	75	5.0
**Relatedness**	Not related	13,010	95.0	13,276	95.1	1,422	99.6	1,479	99.7
	Unlikely	250	1.8	285	2.0	6	0.4	4	0.3
	Related	432	3.2	404	2.9	0	0	1	0.1
**Serious adverse events**		1,428	10.4	1,484	10.6				
**Change of vaccine schedule**	None	13,300	97.1	13,564	97.1	1,383	96.8	1,424	96.0
	Delayed	358	2.6	369	2.6	22	1.5	35	2.4
	Discontinued	34	0.2	32	0.2	23	1.6	25	1.7
**Outcome**	Resolved	13,193	96.4	13,434	96.2	1,231	86.2	1,288	86.8
	Resolved with sequelae	141	1.0	147	1.1	109	7.6	113	7.6
	Unresolved	273	2.0	309	2.2	3	0.2	8	0.5
	Death	85	0.6	75	0.5	85	6.0	75	5.1

A total of 604 volunteers (3.7%; 307 vaccine and 294 placebo recipients), experienced an AE or SAE which was possibly, probably or definitely related to the product in any month after vaccine administration (p = 0.59). The rates of AEs and SAEs attributed to vaccine did not statistically differ between vaccine and placebo recipients.

AEs led to vaccination discontinuation in 66 individuals (0.4%) overall; 34 (0.4%) vaccine and 32 (0.4%) placebo recipients. Eleven of these discontinuations (6 vaccine and 5 placebo recipients) were categorized as related to a product-related AE including angioedema, urticaria, rash/eyelid swelling, headache, syncope (vaso-vagal event) and nephrotic syndrome in the vaccine group and lymphadenitis, lip/eyelid angioedema and rash (n = 3) in the placebo group.

### Serious Adverse Events

A total of 2,912 SAEs (1,428 in vaccine and 1,484 in placebo groups) were reported by 2,394 volunteers (14.6%) with no evidence of a significant difference between vaccine (1,175; 14.3%) and placebo recipients (1,219; 14.9%) (p = 0.33) with an odds ratio for active vaccination of 0.96 (95% CI 0.88–1.04). At 6, 12 and 42 months the estimated SAE rates for the combined treatment arms were 2.8%, 5.2% and 15.8% and a difference between distributions for the times to SAEs were not different by treatment arm. Most SAEs occurred outside the vaccination months with <2% SAE rate during the 30 days post treatment as shown in Table 4. However, female gender was significantly associated with higher SAE frequencies (15.5% in female vs. 13.6% in male vaccine recipients and 16.9% vs. 13.6% in placebo recipients, p<0.001) (Table 5). Both type and frequency of SAEs were similar between vaccine and placebo groups (Table 7). SAEs coded under ‘Injury and procedural related complications’ were the most common type reported followed by those in the ‘Infection and Infestation’ category. More than 90% of the SAEs were graded moderate to potentially life threatening in both groups (p = 0.32). However, 99.9% were scored as not related to the product administration in either group, and <2% discontinued or delayed vaccination. Six SAEs were scored as unlikely related to vaccine administration (influenza at day 169, exacerbation of schizophrenia at day 22, peptic ulcer at day 28, spontaneous abortion at day 309, gastro-intestinal disturbance due to accidental pesticide exposure at day 781, syncope and hematoma on forehead at day 84, and nephrotic syndrome at day 14) while only one SAE (fever at day 26) was scored a possibly related to placebo administration. A majority of participants had SAE resolution (86%) in both groups, while <10% were deaths (Table 6).

**Table 7:** Frequency of common serious adverse events in both vaccine and placebo groups according to system organ class.

	Vaccine	Placebo
	n	n
Injury, poisoning and procedural complication	529	549
Infection and infestation	363	370
Pregnancy and associated conditions	187	194
Gastro-intestinal disorders	103	100
Psychiatric disorders	37	42

#### Deaths

A total of 160 (1.0%) deaths occurred during the study period: 85 vaccine and 75 placebo recipients (p = 0.43). None of the deaths was deemed related to treatment. Overall half (54%) of the deaths in both groups were due to road traffic accidents and trauma-associated events, followed by cardio-vascular causes (8 cases), of which sudden unexplained death syndrome (5 cases: 2 vaccine and 3 placebo recipients) was the most common event.

#### Pregnancy outcomes

A total of 967 (30.6%) vaccine and 955 (30.1%) placebo female recipients reported a pregnancy during the study while 139 vaccine and 116 placebo recipients reported more than one pregnancy (Table 8).

**Table 8:** Frequency of pregnancies and abnormal pregnancy outcomes (APO) in vaccine and placebo groups.

	Vaccine		Placebo		Total		P value
	n	%	n	%	n	%	
Women recipients	3,165	50	3,169	50	6,334	100	
Women with no pregnancy	2,198	69.4	2,214	69.9	4,412	69.7	
Women with at least one pregnancy	967	30.6	955	30.1	1,922	30.3	0.72
Women with one pregnancy	828	26.2	839	26.5	1,667	26.3	
Women with two pregnancies	132	4.2	110	3.5	242	3.8	
Women with three pregnancies	7	0.2	6	0.2	13	0.2	
Pregnancy occurring ≤3 months of last vaccination*	212	21.9	209	21.9	421	21.9	
Pregnancy occurring >3 months of last vaccination*	737	76.2	725	75.9	1,462	76.1	
Overall women with APO	165	5.2	139	4.4	304	4.8	0.13
Pregnant women with APO	165	17.1	139	14.6	304	15.8	0.13
APO ≤3 months of last vaccination	48	22.6	36	17.2	84	19.9	0.18
APO >3 months of last vaccination	105	14.2	92	12.7	197	13.5	0.38
APO in women with first pregnancy	161	16.6	132	13.8	293	15.2	0.09

*Women with known last menstrual period.

Birth was reported for 1,843 infants, 14 of them representing 7 twin pairs. Of these, 277 births (137 vaccine and 140 placebo recipients; 1 twin pair per treatment) occurred within 450 days of study entry. For these infants, birth weight, gestational age and Apgar scores were similar between the vaccine and placebo groups (data not shown). Three congenital abnormalities (1 vaccine and 2 placebo recipients) were reported among these 277 births, the vaccine group abnormality being a respiratory distress syndrome with patent *ductus arteriosus*.

Abnormal pregnancy outcomes (APOs) were experienced in 165 out of 3165 (5.2%) vaccine and 139 out of 3169 (4.4%) placebo female recipients (p = 0.13) and in 17.1% and 14.6% (p = 0.13) of vaccine and placebo pregnancies, respectively. Abnormal outcomes for first pregnancy occurred in 161 (16.6%) and 132 (13.8%) pregnant vaccine and placebo recipients, respectively (p = 0.09). In women with their first pregnancy, the induced abortion rate was 4.9% (94 out of 1922) while the spontaneous abortion rate was 9.1% (175 out of 1922). The overall rate of spontaneous and induced abortions combined was 14% (269 out of 1922) for first pregnancies and 13.6% (297 out of 2190) for all pregnancies.

Women agreed to avoid pregnancy from just prior to the first injection through 3 months following final vaccination as a precaution against adverse outcomes during the most vulnerable stage of fetal development in the first trimester. Because vaccine harm may differ with the period of gestation (early or late) [46], the data were further characterized by time from last vaccination to estimated date of conception. Among pregnancies with estimated dates of conception within 3 months of a vaccination, APOs occurred in 48 of 212 (22.6%) and 36 of 209 (17.2%) vaccine and placebo recipients, respectively (p = 0.18). Among these women, the large majority of these APOs were spontaneous or elective induced abortions with 47 of 212 (22.2%) and 32 of 209 (15.3%) in vaccine and placebo recipients, respectively (p = 0.08). Abnormal pregnancy outcomes among pregnancies with estimated dates of conception greater than 3 months after vaccination were not different between study arms with 105 of 737 (14.2%) and 92 of 725 (12.7%) in vaccine and placebo recipients, respectively (p = 0.38).

## Discussion

The world's first community-based efficacy trial to test an HIV prime-boost vaccine regimen was conducted in 16,402 healthy Thai volunteers, providing the largest safety and reactogenicity data set on a prime-boost regimen with ALVAC-HIV and AIDSVAX B/E. Although the vaccine products (ALVAC-HIV alone or in prime-boost regimens) had been previously evaluated in different populations, the safety data from this study are similar to previous observations in Phase I/II and III studies conducted in Thailand and elsewhere [26], [30], [43], [44], [47]. These safety data may be summarized as follows: most of vaccine recipients experienced either local or systemic reactions significantly more frequently than placebo recipients; the frequency of local reactions such as pain and tenderness were higher than that of systemic reactions such as headache, fatigue, arthralgia and myalgia, although fever was rarely reported; ALVAC-HIV is more reactogenic than AIDSVAX B/E; the frequency of the reactions gradually declined with subsequent vaccine administrations; all local and systemic reactogenicity symptoms were mild to moderate in nature, resolving rapidly and spontaneously in the vast majority of cases.

Routine biochemistry and hematologic laboratory values were not assessed in RV144 based on the safety profile observed in previous Phase I and II studies. No differences in any parameter of renal function, hematologic abnormalities, or alterations in CD4 T-cell count were noted among recipients of ALVAC-HIV alone, recipients of ALVAC-HIV with a subunit vaccine, or recipients of control [30].

The number of cases identified as AEs fall into the usual broad categories that were previously described in phase I/II studies. The frequency of AEs was not significantly different between vaccine and placebo groups. Most AEs occurred after the 30-day post-vaccination period, 3.2% of AEs and none of SAEs being attributed to vaccine. Female participants experienced a higher frequency of AEs and SAEs in both vaccine and placebo groups. The reasons for this difference are unclear. In other studies, male participants experienced less pain than female participants following ALVAC-HIV administration [30]. Although a fair comparison cannot be established with Phase I/II trials of the same vaccination regimen, their duration of follow-up being considerably shorter, these observations are in agreement with previous reports that there are few AEs and no SAE related to the administration of this vaccine. In the AIDSVAX B/E phase III trial conducted in 2546 injecting drug users (mostly male) in Bangkok, the proportion of SAEs reported (414, 16.2%) did not differ between vaccine and placebo groups and was similar to the 14.6% reported in this study [29].

Although ALVAC-HIV is not a vaccinia-derived vaccine, none of the vaccinated individuals presented post vaccination symptoms suggestive of myopericarditis events as described after smallpox vaccination [48], [49].

None of the 160 deaths reported in this trial were assessed as related to the candidate vaccines and were mostly related to trauma and cardiovascular causes. The number of Sudden Unexplained Death Syndrome (SUDS) events (n = 5) is less than the expected case number (8.4) calculated from the number of person years (32,300 male person years) and the published rate estimate in 20–49 year old men from northeastern Thailand (25.9/100,000 person years) [50]. In a previous AIDSVAX B/E efficacy trial conducted in 2527 injecting drug user participants in Bangkok, 102 deaths were reported with no difference between vaccine and placebo recipients and none being attributed to vaccine [29]. A similar observation was made on phase I/II trials of ALVAC alone or ALVAC and subunit prime-boost regimens with 7 deaths out of 1497 participants, none related to vaccination [30].

Overall, the prime-boost regimen did not result in more abnormal pregnancy outcomes in vaccine than in placebo female recipients. This corroborates previous findings in phase I/II and III trials [29], [30]. In Thailand, the induced abortion ratio has been estimated at 19.5 for 1000 live births [51] contrasting with the 4.9% reported in this study. In this study, the proportion of induced abortions documented must be interpreted with caution, as induced abortion is illegal in Thailand and most pregnancy outcome data are derived from the volunteer's report. The spontaneous abortion rate of 9.1% is closer to the estimated rate of 6.9% formerly reported from a Thai hospital [52]. Although not statistically significant, the higher number of abortions (spontaneous and induced) among vaccine recipients merits close scrutiny in future trials of ALVAC and protein combinations.

Small clinical trials with either recombinant canarypox or envelope subunit vaccines did not reveal safety issues in newborns and infants from HIV-infected mothers [53]–[55]. Moreover, gp120 envelope subunit was shown to be safe in HIV-infected pregnant women [56]. In several studies, ALVAC-HIV (vCP1452) has been safely administered to immuno-compromised HIV-infected subjects [57]–[59]. ALVAC recombinants have been administered to humans and animals by parenteral and oral routes without signs of replication, systemic dissemination or severe reaction. In principle, it should be impossible for canarypox recombinants to disseminate in the environment, as the recombinants would not be synthesized in mammalian cells as complete virus. ALVAC is an attenuated canarypox virus and is non-pathogenic in its host species, other birds, mammals and humans. It may be infectious for birds, though there are already five canarypox-based veterinary vaccines [60].

The results of the ALVAC-HIV and AIDSVAX B/E prime-boost regimen confirm that the regimen is safe and well tolerated among a large population of healthy HIV-uninfected adults in Thailand. Although occurrence of local and systemic reactions was reported among the vaccinated participants, very few adverse events were related to the vaccine products. No death was attributed to the vaccination regimen. Altogether, these findings indicate that ALVAC-HIV and AIDSVAX B/E are safe and well tolerated and may be suitable for further study and large-scale public use, should efficacy be judged adequate to have a public health impact.

## Supporting Information

Checklist S1 CONSORT Checklist.
(DOC)

Protocol S1 Trial Protocol.
(DOC)
